# Interlaboratory Comparison of Magnetic Thin Film Measurements

**DOI:** 10.6028/jres.108.012

**Published:** 2003-04-01

**Authors:** F. C. S. da Silva, C. M. Wang, D. P. Pappas

**Affiliations:** National Institute of Standards and Technology, 325 Broadway, Boulder, CO 80305

**Keywords:** interlaboratory comparison, magnetic films, standard reference materials

## Abstract

A potential low magnetic moment standard reference material (SRM) was studied in an interlaboratory comparison. The mean and the standard deviation of the saturation moment *m*_s_, the remanent moment *m*_r_, and the intrinsic coercivity *H*_c_ of nine samples were extracted from hysteresis-loop measurements. Samples were measured by thirteen laboratories using inductive-field loopers, vibrating-sample magnetometers, alternating-gradient force magnetometers, and superconducting quantum-interference-device magnetometers. NiFe films on Si substrates had saturation moment measurements reproduced within 5 % variation among the laboratories. The results show that a good candidate for an SRM must have a highly square hysteresis loop (*m*_r_/*m*_s_ > 90 %), *H*_c_ ≈ 400 A·m^−1^ (5 Oe), and *m*_s_ ≈ 2 × 10^−7^ A·m^2^ (2 × 10^−4^ emu).

## 1. Introduction

Control of a wide range of magnetic properties is critical in the manufacturing of magnetic data-storage devices. These properties include, among others, the saturation and remanent magnetic moments, and the intrinsic coercivity. The desired values for these properties are specific to the particular application and can cover a wide range. This is illustrated in a data storage system, based on magnetic tape or hard disk drives, where thin films with low intrinsic coercivities are used in the read heads while relatively thick films with high moments and high intrinsic coercivities are used for the storage media. These devices can involve multiple layers of various magnetic and nonmagnetic materials composed of an assortment of alloys.

In addition, an important problem in the technology and manufacturing of magnetic thin film devices is the determination of the film thickness. This goes beyond the element-specific (e.g., Fe, Ni, and Co) thickness calibration because the relevant properties of devices using this technology are dominated by the interfaces. These properties are affected by intermixing and alloying, pinholes, reduced atomic coordination, and quantum-well effects. These effects are in turn determined by growth temperature, composition modulation, substrate strain, and microscopic morphology of the films. Therefore, it is necessary to measure the actual magnetic properties of samples as deposited in order to correlate useful properties (magnetoresistance, intrinsic coercivity, anisotropy) with magnetic moments. Recent developments in technology that use magnetic layers less than 10 nm thick, e.g., giant magnetoresistance (GMR) sensors with correspondingly low moments (on the order of 10^−8^ A·m^2^), present stringent requirements on process and quality control.

Therefore the calibration of magnetic-property measurement techniques at the lowest range is important for process and quality control as well as for research. Because most of the measurement techniques currently in use are sensitive to the fields generated by the sample, they are also sensitive to the sample geometry. Hence, care must be taken to choose a calibration reference artifact that has both a small moment and the same form factor (thin film geometry) as the samples to be measured. At present, NIST offers two standard reference materials (SRMs) for magnetometer calibration: SRM 762 and SRM 772a. Both SRMs have magnetic moments in the scale of 10^−3^ A·m^2^ (1 emu).

The objective of this study was to investigate the calibration needs of the magnetic recording industry and identify the greatest need for standard reference samples. This required evaluating several measurement techniques, magnetic properties, and samples. We concentrated on a few common measurement techniques and low moment samples, similar to those used in magnetoresistive read heads.

## 2. Methods

An interlaboratory comparison study for magnetic characterization was undertaken in which 9 ferromagnetic samples were sent to 13 laboratories from the magnetic recording industry, NIST, academia, and magnetic instrument manufacturers. The samples were circulated in a serial fashion, with each laboratory allotted approximately 3 days for measurements. Table 1 shows the composition and dimensions of the 9 samples.

Two different types of samples were prepared. The first type was composed of a Permalloy (Ni_81_Fe_19_) film sandwiched between Ta layers. These samples are similar to the free magnetic layer used in magnetoresistive (MR) heads in terms of thickness, magnetization, total moment, intrinsic coercivity, and geometry. Wafers 7.5 cm in diameter were used for samples A, B, and C because the first step in the head manufacturing process requires quality control at the wafer level. Smaller sizes were used for samples D, E, and F because the second step in quality control is typically to dice wafers and study them at the coupon level with high-field magnetometers. These two sample geometries allowed us to make direct connections to relevant processes in head metrology. The second type of samples were ultra-thin single-crystal Ni films grown on diamond substrates and capped with Cu. These samples have well characterized magnetic and structural properties [1–7] and moments comparable to those of samples used for head metrology.

Four types of magnetic measurement tools were used in this study: vibrating-sample magnetometer (VSM) [8,9], alternating gradient force magnetometer (AGM) [10–12], superconducting quantum-interference-device (SQUID) magnetometer [13], and inductive-field (B-H) looper [14]. In practice, these tools fall into two general categories: magnetometric and fluxmetric. In magnetometric systems (AGM, VSM, and SQUID), the magnetic field generated by the sample (approximated by a dipole) is measured. In fluxmetric systems (B-H loopers) the fields inside the sample are measured by directly measuring the flux variations around a cross section of the sample.

The tools in the first category (VSM, AGM, and SQUID) are generally used with small samples (dimensions on the order of 1 cm) that are suspended on a long, slender rod. The rod is adjusted so the sample is positioned near a relatively large pickup loop or modulating coil. This category of magnetometers has the advantage that the sample can be placed between the poles of an electromagnet. However, the physical position, sample size, and mounting procedures are very important. If these problems are addressed correctly, this first category can be calibrated in terms of the total moment of the sample.

In the second category of tools (B-H loopers), the sample is held in a rigid fixture inside a coreless magnetizing coil with a relatively short, close-fitting inductive sense coil. This reduces the sensitivity of the measurement to sample alignment. In general, B-H loopers are useful for thin-film samples of large area and are used to screen entire wafers in quality control. In this geometry, the measured quantity is the flux enclosed by the sense coil.

Without making assumptions about sample shape, homogeneity, or field distribution, it is not possible to directly analyze the results of the two categories of measurement tools. In this study, therefore, we focused on the consistency between relative measurements of two different samples. For calibrated measurements, measured voltages have to be scaled by the voltage corresponding to a standard reference material. This means that the absolute quantities reported in this paper reflect the reproducibility of the laboratories’ own measurements.

A form was sent with the samples to ensure uniform reporting of results. Information to be entered included the laboratory, operator, date, sample measured, and technique used. The section for the results requested measurements of the total saturation moment *m*_s_, total remanent moment *m*_r_, and intrinsic coercivity *H*_c_. Finally, the back side of the form contained instructions for handling the sample and a table for the operator to record the measurement parameters (e.g., maximum field, sweep rates, integration time constants.)

## 3. Results

**Fig. 1:**
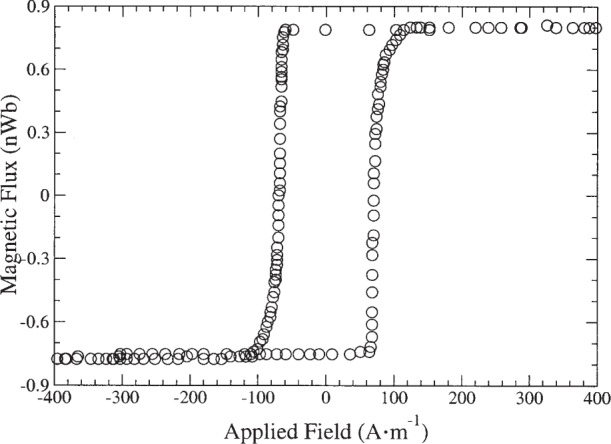
Hysteresis curve of sample A measured with a B-H looper.

**Fig. 2:**
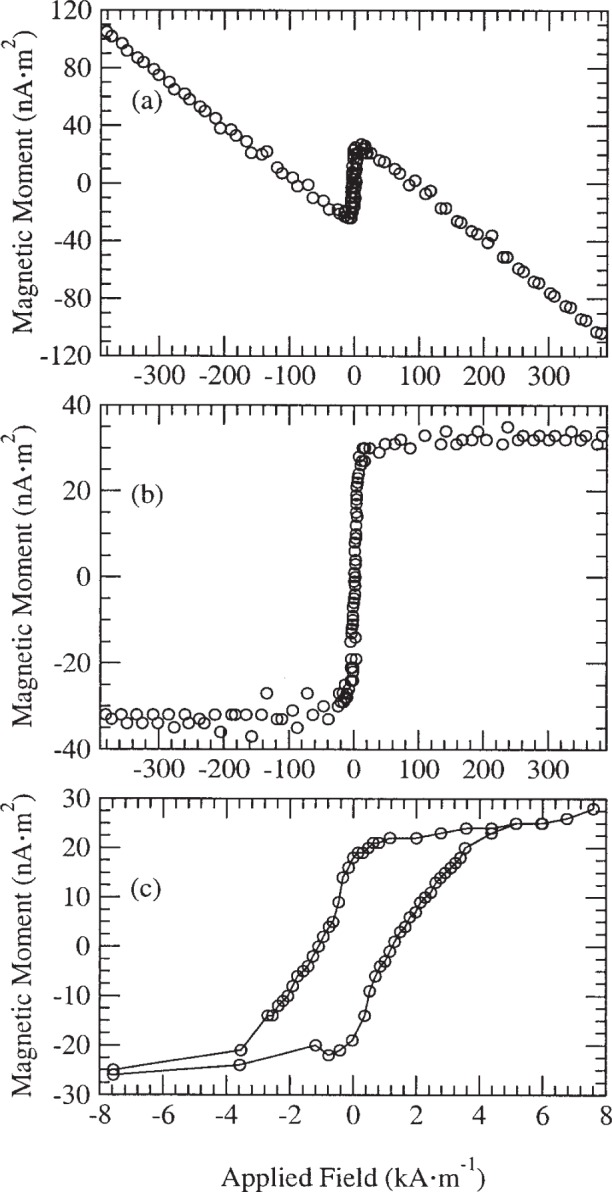
Hysteresis curve of sample H measured with a SQUID, showing the data (a) before and (b–c) after the diamagnetic subtraction.

**Fig. 3:**
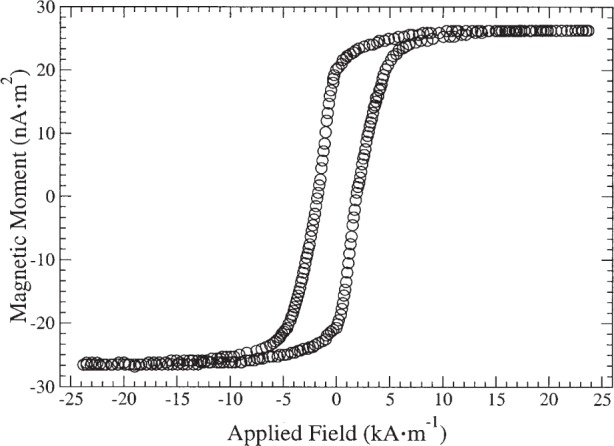
Hysteresis curve of sample H measured with an AGM.

**Fig. 4:**
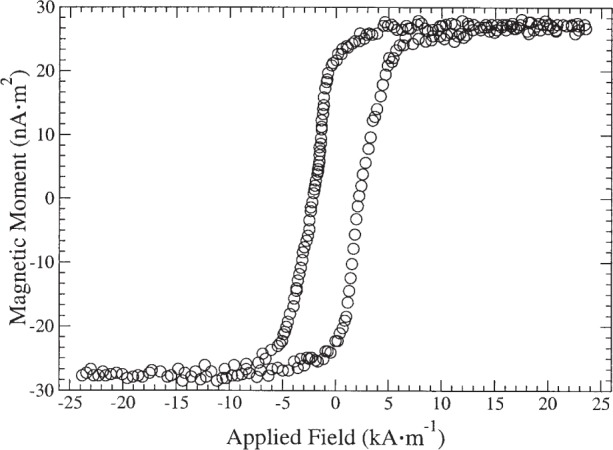
Hysteresis curve of sample H measured with an VSM.

Figures 1, 2, 3, and 4 show examples of hysteresis curves obtained by the participating laboratories using the four different measurement techniques. For all curves, the signal-to-noise ratio (*SNR*) was obtained by taking the amplitude of the total curve (≈ 2*m*_s_) and dividing by the standard uncertainty of the noise after saturation $\left[\sqrt{\langle \delta^{2}m\rangle }\right]$. The estimates of uncertainties for *m*_s_ and *m*_r_ were defined as the reciprocal of the *SNR*.

Figure 1 shows a hysteresis curve measured on sample A using a B-H looper. Here, the measurement was performed at a field frequency of 2 Hz with 10 averages used to obtain the final curve. Notice that the magnetic flux *Φ* is reported instead of the magnetic moment *m*. This did not affect the comparison between samples A, B, and C since they were measured only with B-H loopers. Since *SNR*_BH_ = 1000 for Fig. 1, we report the fixed uncertainty in *m*_s_ and *m*_r_ for B-H loopers as 0.1 %.

Figure 2(a) shows the hysteresis curve of sample H measured with a SQUID magnetometer. Here, the time interval between points on the curve was 200 s. The raw data show the diamagnetic contribution due to the diamond substrate. This dramatic effect is a combination of the diamagnetic susceptibility of diamond, which is 50 % greater than that of Si, and the low mass ratio between the Ni film and the diamond substrate. The result after the subtraction of the diamagnetic contribution is shown in Fig. 2(b), from which we obtain *SNR*_SQUID_ = 16. This *SNR* gives an uncertainty in the magnetic moment of 6.3 %. Figure 2(c) shows the same corrected curve where better resolution around zero applied field leads to estimates of the remanence and intrinsic coercivity.

Figure 3 shows a hysteresis curve of sample H measured with an AGM. The time between points in this measurement was 1 s. The curve does not show the diamagnetic contribution because lower magnetic fields were applied. Here, *SNR*_AGM_ = 54, giving an uncertainty in the magnetic moment of 1.9 %.

Figure 4 shows the hysteresis curve of sample H measured with a VSM. At an interval of 1 s between measurements, we have *SNR*_VSM_ = 15, yielding an uncertainty of 6.7 % in the magnetic moment.

**Fig. 5:**
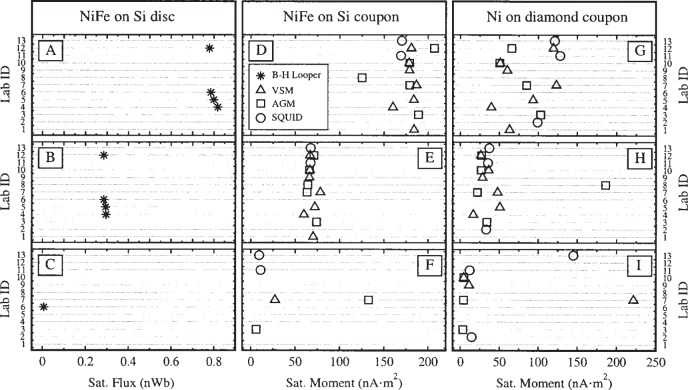
Interlaboratory comparison data showing the absolute values of the saturation moment *m*_s_. B-H looper measurements of flux on samples D and E are shown in Table 2.

**Fig. 6:**
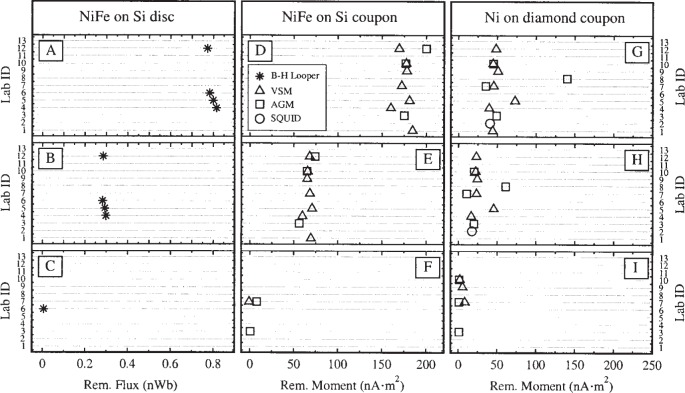
Interlaboratory comparison data showing the values of the remanent moment. B-H looper measurements of flux on samples D and E are shown in Table 3.

**Fig. 7:**
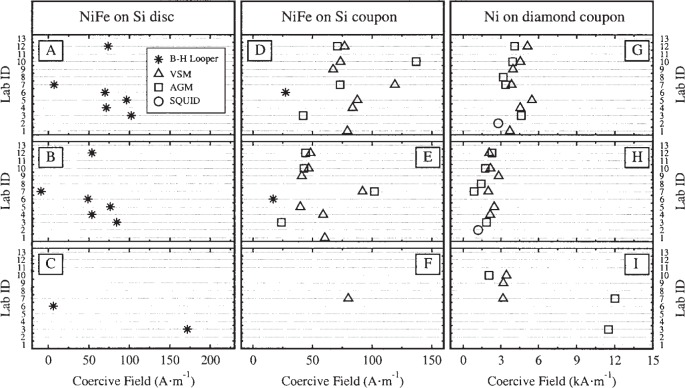
Interlaboratory comparison data showing the values of the intrinsic coercivity.

The hysteresis curves were used to extract three quantities: the saturation moment *m*_s_, the remanent moment *m*_r_, and the intrinsic coercivity *H*_c_. Figures 5, 6, and 7, respectively present the measured values of *m*_s_, *m*_r_, and *H*_c_ for all nine samples and all four measurement techniques. Some samples were measured more than once by the same laboratory using the same technique. In these cases, the reported quantity is the average of two measurements of the same quantity. All SQUID measurements, on the other hand, provided only one measurement of *m*_s_ and *m*_r_.

A simple rule for outlying points was based on the report of each laboratory on how the measurements were done. A few measurements were discarded when the calibration procedure did not follow the procedure used by the other laboratories. However, all relative measurements (e.g., *m*_s_/*m*_r_ ratio) could be used since the effect of calibration factors was minimized.

**Fig. 8:**
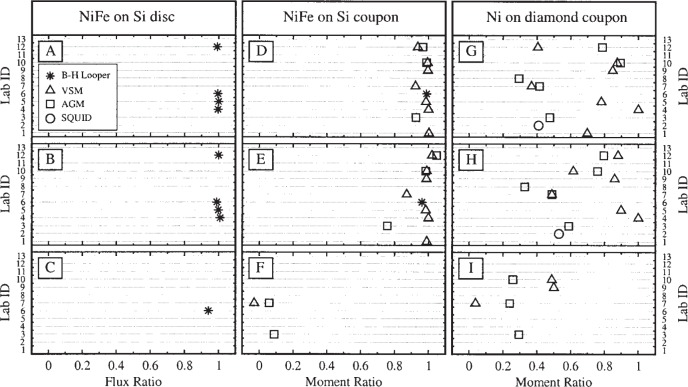
Interlaboratory comparison data showing the remanence-to-saturation ratio for each sample.

**Fig. 9:**
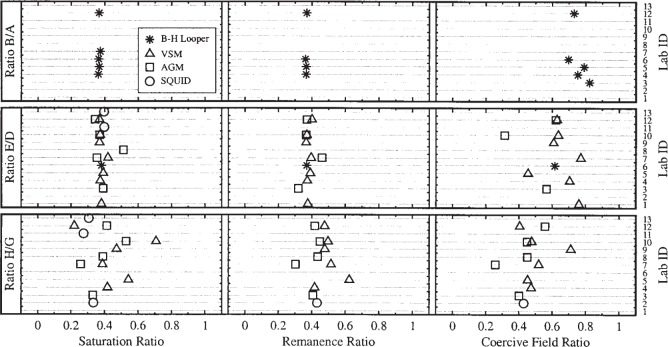
Interlaboratory comparison data showing the intralaboratory ratios of the saturation moments, the remanent moments, and the intrinsic coercivities between two samples.

Figure 8 shows the ratio *m*_s_/*m*_r_ obtained using the data shown in Figs. 5 and 6. Absolute B-H looper measurements on samples D and E could not be compared with the other techniques for lack of an unambiguous procedure to convert from magnetic flux to magnetic moment. However, it was still justifiable to compare the ratios *m*_s_/*m*_r_ and $m_{s}^{\text{SampleE}}/m_{s}^{\text{SampleD}}$ (Figs. 8 and 9), since the relative conversion factors cancel.

Each absolute quantity measured for a given sample using a particular technique by different laboratories formed a set. The mean and the standard uncertainty of each set were calculated. The reproducibility parameter here was calculated as the standard uncertainty of the quantities in each set. Tables 2–4 list the means and the standard uncertainties of the saturation and remanent moments and the intrinsic coercivity across the laboratories. The ratio of the remanent-to-saturation moment of each sample and the saturation moment ratio between samples is shown in Tables 5 and 6.

## 4. Discussion

The main goal of this interlaboratory comparison was to identify standard procedures and materials that could provide reproducible laboratory measurements. Due to a few non-conformities, a precise analysis of the statistical data [15] was not possible. The specified measurement procedures were not followed exactly by all the participants. Also, not all quantities requested in the form were measured twice to provide uncertainties to the measurements. In the laboratories’ reports, samples F and I showed initial stages of oxidation, which compromises any conclusion about these samples. However, the data showed enough statistical validity to determine the main features for a low magnetic moment SRM candidate.

Magnetic flux for samples A, B, and C (Permalloy on Si wafer) could be measured with an uncertainty of 3 %. This result shows that SRMs in the shape of wafers are useful for measurements of saturation and remanence. A procedure similar to that used for calibrating magnetometric measurement systems needs to be developed in order for comparisons (after a wafer dicing, for example) to be done in moment units.

The diamagnetism in some reported magnetometric hysteresis curves is an undesired source of uncertainty. Since low magnetic moment samples are usually thin films on bulk substrates, SRMs have to be specified with low intrinsic coercivity to minimize diamagnetic effects due to the substrate. Also, to ensure that the magnetic film is a monodomain in the saturated state, the SRMs have to be specified with high squareness (*m*_r_/*m*_s_ ≈ 1). These specifications were confirmed by saturation measurements performed on samples D and E (Permalloy on Si coupon), which showed mean standard uncertainties of 8 % and 5 %, respectively.

The measurements of intrinsic coercivity show the most scattered data. Although no environmental conditions were reported, we know that the measurements were likely affected by temperature, humidity, measurement time constants, and field uncertainties.

## 5. Conclusions

The data presented show a path to the production of a low magnetic moment standard reference material. For fluxmetric systems, a round sample of Permalloy on a Si wafer seems to be a good candidate, with an estimated interlaboratory standard uncertainty of saturation flux of 3 %. For magnetometric systems, a possible candidate must have a highly square hysteresis loop (*m*_r_/*m*_s_ ≈ 1), *H*_c_ of about 400 A·m^−1^ (5 Oe), and *m*_s_ ≈ 2 × 10^−7^ A·m^2^ (2 × 10^4^ emu). Such an SRM can be made out of Permalloy films on Si substrates, which showed the best estimated interlaboratory standard uncertainty of the saturation moment of 5 %.

## Figures and Tables

**Table 1:** Samples used in the study

Sample	Composition	Nominal magnetic film dimensions
A	Si/Ta/NiFe/Ta	π × (38 mm)^2^ × 10 nm
B	Si/Ta/NiFe/Ta	π × (38 mm)^2^ × 5 nm
C	Si/Ta/NiFe/Ta	π × (38 mm)^2^ × 2.5 nm
D	Si/Ta/NiFe/Ta	5 mm × 5 mm × 10 mm
E	Si/Ta/NiFe/Ta	5 mm × 5 mm × 5 mm
F	Si/Ta/NiFe/Ta	5 mm × 5 mm × 2.5 mm
G	C(100)/Ni(100)/Cu(100)	3 mm × 2 mm × 25 mm
H	C(100)/Ni(100)/Cu(100)	3 mm × 2 mm × 10 mm
I	C(100)/Ni(100)/Cu(100)	3 mm × 2 mm × 2.5 mm

**Table 2:** Statistical analysis of the saturation moment. In each cell, the top values are the mean and the bottom values are the standard uncertainties divided by the corresponding means

Saturation flux (pWb)									
Method	A	B	C	D	E	F	G	H	I
BH	795.3	290.1	6.8	55.5	21.1				
	2.2 %	1.8 %							

Saturation moment (nA·m^2^)									
Method	A	B	C	D	E	F	G	H	I
VSM				179	68	27	78	34	79
				5 %	9 %		44 %	38 %	160 %
AGM				176	68	69	76.0	59	4
				17 %	7 %	130 %	30 %	120 %	15 %
SQUID				169.5	67.2	10.2	83.0	24.1	52.3
				0.4 %	0.1 %	12 %	87 %	87 %	150 %

**Table 3:** Statistical analysis of the remanent moment. In each cell, the top values are the mean and the bottom values are the standard uncertainties divided by the corresponding means

Remanent flux (pWb)									
Method	A	B	C	D	E	F	G	H	I
BH	792.0	289.9	6.4	54.9	20.3				
	2.4 %	2.4 %							

Remanent moment (nA·m^2^)									
Method	A	B	C	D	E	F	G	H	I
VSM				175	67		49	26	5
				4 6 %	6 %		22 %	38 %	60 %
AGM				183.9	65.0	4.2	45.2	18.1	1.0
				76 %	14 %	120 %	16 %	28 %	1 %
SQUID							40.5	17.5	

**Table 4:** Statistical analysis of the intrinsic coercivity. In each cell, the top values are the mean and the bottom values are the standard uncertainties divided by the corresponding means

Intrinsic coercivity (A·m^−1^)									
Method	A	B	C	D	E	F	G	H	I
BH	70	51	86	28	17				
	49 %	65 %	27 %						
VSM				84	55	80	4451	2277	3272
				20 %	35 %		15 %	14 %	5 %
AGM				81	53	1576	3836	1637	8527
				50 %	60 %	110 %	15 %	32 %	66 %
SQUID							2785	1194	

**Table 5:** Statistical analysis of the remanent-to-saturation moment ratio. In each cell, the top values are the mean and the bottom values are the standard uncertainties divided by the corresponding means

Remanent to saturation moment ratio									
Method	A	B	C	D	E	F	G	H	I
BH	0.996	0.999	0.941	0.988	0.962				
	0.3 %	0.8 %							
VSM				0.98	0.98		0.71	0.79	0.26
				0.3 %	0.5 %		34 %	25 %	100 %
AGM				0.73	0.70	0.07	0.57	0.59	0.26
				64 %	67 %	29 %	46 %	32 %	12 %
SQUID							0.41	0.53	

**Table 6:** Statistical analysis of the ratio of magnetic quantities of two samples. In each cell, the top values are the mean and the bottom values are the standard uncertainties divided by the corresponding means

Saturation ratio Remanence ratio Intrinsic coercivity ratio									
Method	B/A	E/D	H/G	B/A	E/D	H/G	B/A	E/D	H/G
BH	0.37	0.38		0.98	0.37		0.76	0.62	
	3 %			140 %			7 %		
VSM		0.38	1.75		0.38	0.43		0.65	0.43
		5 %			3 %	50 %		17 %	51 %
AGM		0.39	2.87		0.38	0.40		0.72	0.42
		18 %	138 %		16 %	15 %		65 %	26 %
SQUID		0.40	3.59			0.43			0.43
			15 %						
